# Transhepatic approach for rehabilitation of stenosed pulmonary arteries

**DOI:** 10.4103/0974-2069.64369

**Published:** 2010

**Authors:** Makram R Ebeid

**Affiliations:** Department of Pediatrics (Division of Pediatric Cardiology), University of Mississippi Medical Center, Mississippi, USA

**Keywords:** Congenital heart disease, interventional catheterization, occluded vessels, pulmonary angioplasty

## Abstract

Transhepatic cardiac catheterization and intervention is used in selected cases in our institution. A retrospective review of transcatheter interventions for the pulmonary artery was conducted. Forty-five transhepatic procedures were performed. Thirteen involved intervention, to rehabilitate the branch pulmonary arteries. The median weight of the patients was 9.9 Kg ± 3.4. The patients' age ranged from eight months to 86 months (median 23 months). The largest sheath used was 7F. All the patients underwent success intervention with no complication related either to the transhepatic approach or the intervention. The branch pulmonary artery diameter increased from 4.5 ± 2.2 mm to 7 ± 3 mm. Most of the procedures were performed under conscious sedation / deep sedation protocol. Hemostasis was achieved in all patients by gradual sheath withdrawal, followed by application of upward pressure on the tract from the subcostal area. In the absence of patent femoral veins the transhepatic approach can be used to perform successful and safe interventions, to rehabilitate the pulmonary artery system. It may offer the additional advantage of using larger sheaths than would be felt appropriate for the femoral veins.

## INTRODUCTION

**Figure 1:**
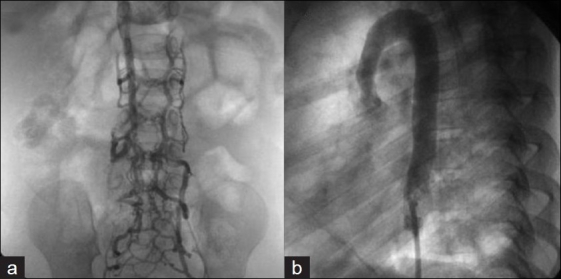
Angiogram shows occluded femoral veins in one patient (a) and interrupted IVC in another patient (b)

As many more patients undergo early surgical interventions as well as cardiac catheterizations, frequently the femoral veins are utilized for access. This results in occlusion of the femoral veins in a number of patients [Figure 1a]. In other instances, anatomical issues such as the interruption of the inferior vena cava may preclude using the femoral veins to perform the required procedure [Figure 1b]. Alternative routes such as the subclavian or the internal jugular vein have been used. Although these are useful routes, they may not allow easy access to the pulmonary arteries for intervention. The transhepatic route is an attractive alternative, which should be considered in such cases to perform pulmonary artery rehabilitation.[1-7]

## TECHNIQUE

**Figure 2:**
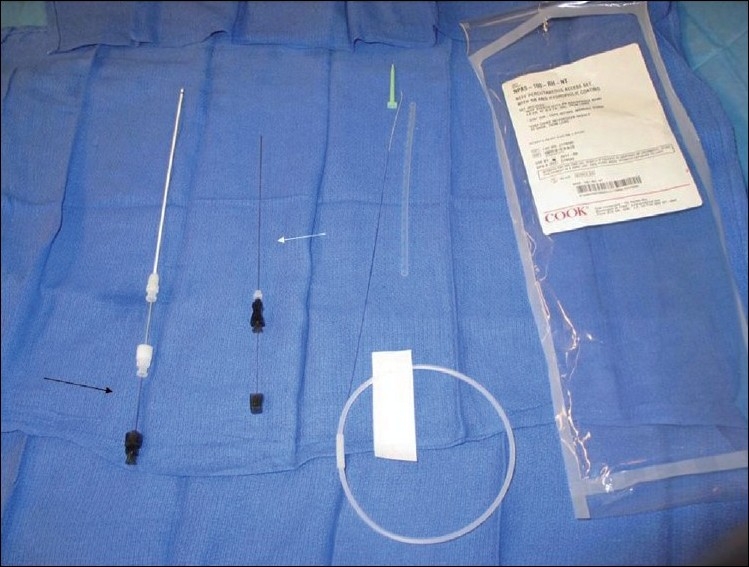
The NEFF set: shows the transitional dilator with a hollow stainless steel wire (black arrow) and the Chiba needle with the obturator (white arrow)

**Figure 3:**
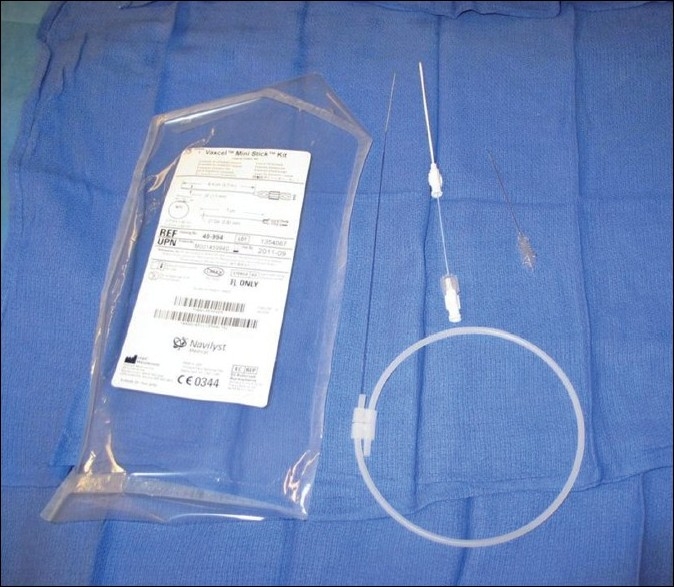
The Mini-Stick Kit™ is shown with its 7 cm needle, transitional sheath, and .021 inch wire

**Figure 4:**
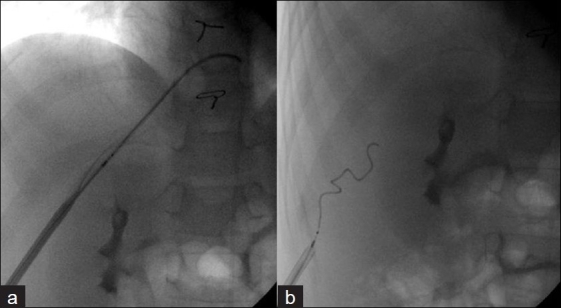
Note needle being directed superior, medial, and posterior as seen in the anterior–posterior (a) and lateral projections (b)

**Figure 5:**
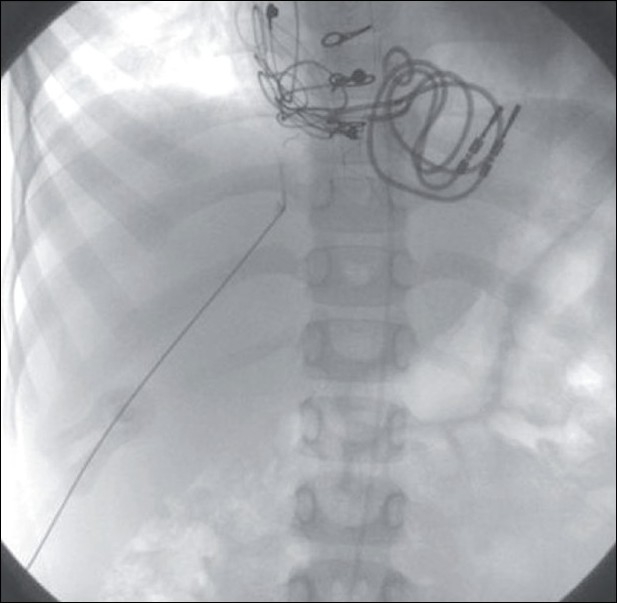
The needle is advanced slightly higher than usual because of the superior position of the liver

**Figure 6:**
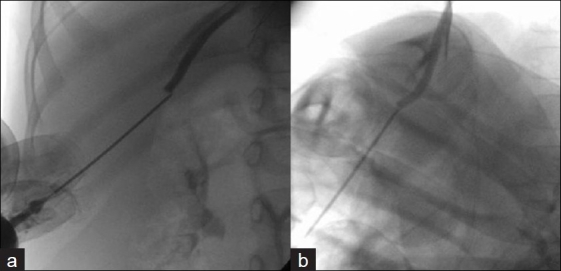
Anteroposterior (a) and lateral (b) projections of the needle while acquiring an angiogram outlining a favorable hepatic vein to be used for placing the sheath. Note the position of the needle in the subcostal region and the straight course of the hepatic vein

**Figure 7:**
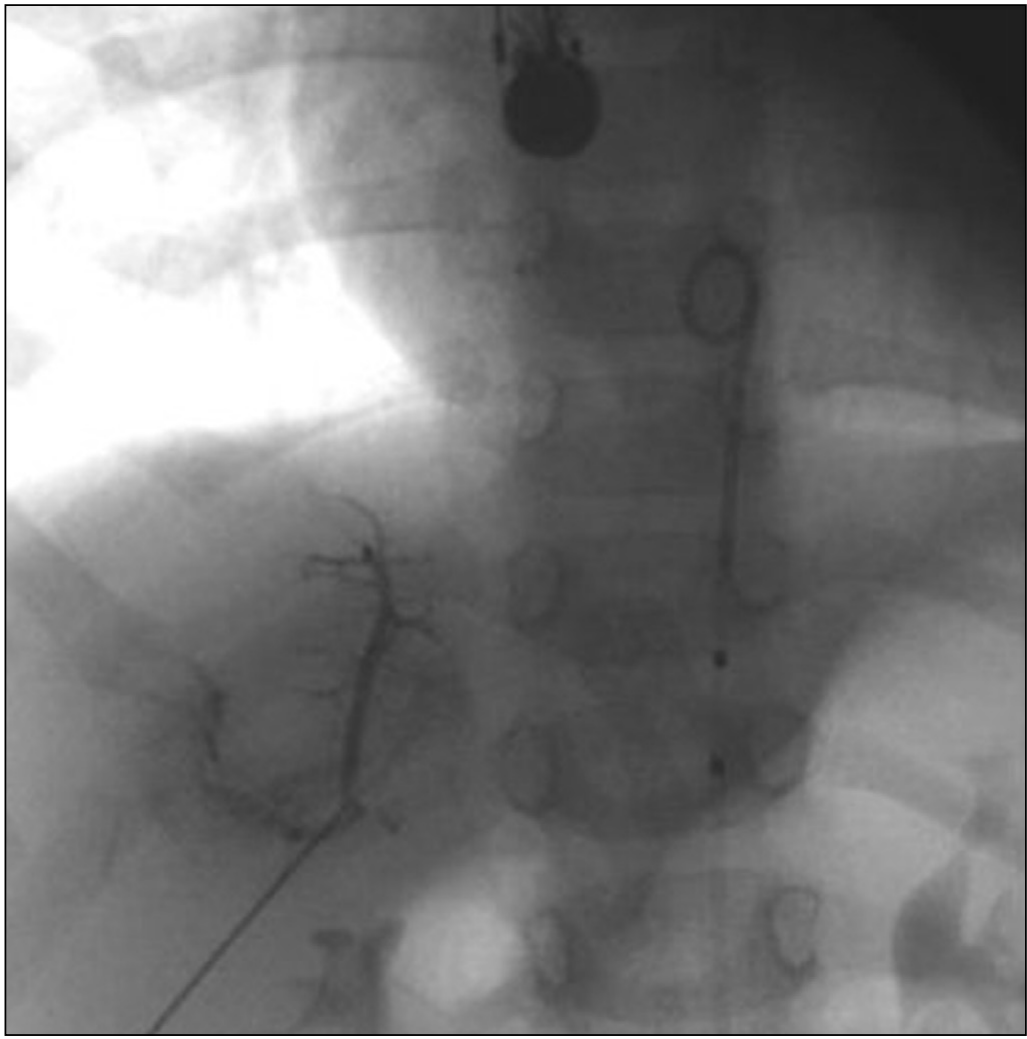
Hand injection outlines a portal vein rather than a hepatic vein

**Figure 8:**
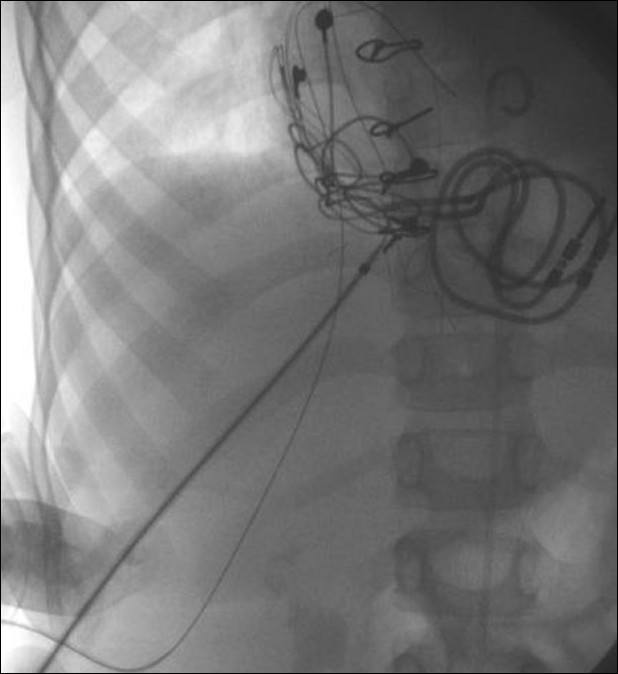
The stiff transitional sheath from the NEFF set can be used with or without the stainless steel wire to upsize the .018 inch wire

To safely perform transhepatic cardiac catheterization, understanding the liver anatomy is important. It is formed by 50,000 - 100,000 hepatic lobules.[8-10] Each lobule contains a central hepatic vein draining to the inferior vena cava surrounded by the hepatic triad (portal vein, bile canaliculus, and the hepatic artery). The target vessel is the hepatic vein leading to the inferior vena cava. The technique that has been previously described[1-4] continues to evolve as more experience and expertise is gained in this approach.[11-15] There are important details that warrant emphasis. The procedure is preferably performed under bi-plane fluoroscopy guidance, although it can be performed using a single plane. It can be safely performed under conscious / deep sedation or general anesthesia. If conscious sedation is to be used the area around the point of entry should be well anesthetized deep into the capsule and the liver parenchyma, as advancing the sheath can be painful. A long 21 or 22 gauge needle is used (with or without an obturator). These are available commercially from many companies. The most commonly used is the Chiba needle (Cook, Blomington, IN) (with or without an obturater), which is 15 cm long [Figure 2]. The Mini-Stick Kit™ (Navilyst Medical, Glenn Falls, NY) system can be used in smaller children . It has a shorter needle (7 cm) and softer transitional dilator [Figure 3] We have generally preferred the latter needle if it reaches at least 2 cm from the midline; usually if the child is < 10 Kg. However in some instances when the latter needle was not stiff enough the Chiba needle has been used with caution. An alternative system includes the Prelude™ transradial system (Merritt Medical. South Jordan, UT). The needle is chosen based on the patients size and distance the needle needs to cross. The Chiba needle is the stiffest and longest. The Neff set (Cook) provides the Chiba needle, transitional dilator, and a stiff hollow wire [Figure 2]. They all have .018 inch wires, but are relatively short and floppy for the hepatic parenchyma. We prefer to use a Road runner wire (.018 in caliber), (Cook), which has a very floppy tip and a stiff body, and is sufficiently long to allow placing the transitional dilators with the stiff part of the wire in the hepatic parenchyma. The preferred area of entry is the subcostal area in the midaxillary line. The needle is directed superiorly, posteriorly, and medially and advanced under fluoroscopy guidance toward the midline (to stop approximately 1 - 1.5 cm from the midline [Figure 4]. In some instances, where the liver is just at the tip of the costal margin, the subcostal area may not be appropriate because of the difficulty in advancing the sheath over the wire, as it will need to take a sharp angle superiorly. This can complicate introducing the sheath.[4] In these instances, an area, one or two intercostal spaces higher can be utilized [Figure 5]. The entry should be below the diaphragm. Likewise, the midaxillary line may not allow easy advancement of the sheath because of the sharp turn it may take to get into the inferior vena cava posteriorly. An alternative site would be between the lateral to midclavicular line with a more acute angle of the needle entry. Occasionally, a pop is felt while introducing the needle. This is a sign that the needle is in a vascular space. If used, the obturator is removed. A syringe with dye is attached to the hub of the needle and gentle aspiration is done until blood is aspirated. Once blood is aspirated, a hand injection with contrast is performed and recorded [Figure 6]. This outlines the vascular system. Prior to advancing the wire, it should be confirmed that the vascular space is indeed the hepatic vein. Occasionally a portal vein or the hepatic artery can be entered [Figure 7]. If either is entered, usually the hand injection would outline the nearby hepatic vein. Guided by the acquired image, the needle can be removed and directed toward the hepatic vein. If the hepatic artery or portal vein is entered and recognized before advancing the sheath, it is usually self-limiting with no sequel. Once the hepatic vein entry is established, a .018 inch long wire is advanced as far as the anatomical substrate allows the stiff part of the wire to be placed, along the tract. The needle is then removed, with the wire maintained in position. Usually, a transitional dilator is used to allow placing a stiffer wire of a larger caliber to facilitate placement of the required sheath.[16] The NEFF set, in addition to the transitional sheath, provides a very stiff, stainless steel, hollow wire (.018 in inner lumen), which straightens the transitional dilator, during advancement, over the .018 wire [Figure 8]. The stainless steel wire does not protrude beyond the dilator but extreme care should be done to avoid shearing of the floppy wire tip. This stiffness provided by the stainless steel hollow wire is occasionally needed to straighten and facilitate advancing the sheath and the dilator along tortuous tracts. It is, however, quite stiff and should be used with extreme caution. The transitional sheath provided with the Mini-Stick is softer and more flexible. It is more appropriate for younger infants. Depending on the planned procedure and the sheath required, the .018 inch wire may be left in place and a small sheath advanced over the wire. Alternatively, if a larger sheath is necessary for the procedure, the transitional sheath would facilitate placing a larger stiffer wire. In rare instances despite advancing the wire it may be difficult to advance the sheath because the wire may have entered through a sharp curve along the capsule inferiorly. In these instances a higher entry point, while staying below the diaphragm, is helpful. The wire can be kept in place to guide as a target.

## CATHETER MANIPULATION

**Figure 9:**
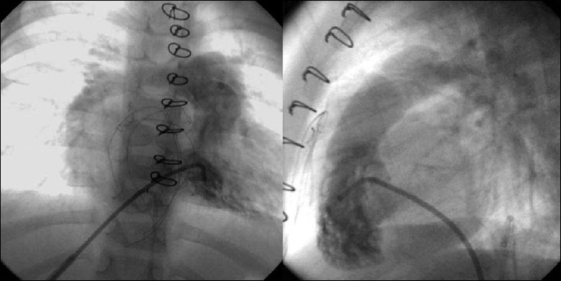
A Judkins right catheter facilitates entry to the anterior right ventricle

**Figure 10:**
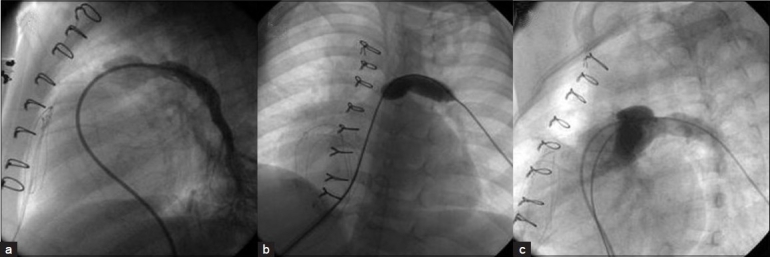
Balloon angioplasty of the left pulmonary artery. Shows pre-balloon (a), during balloon (b) and post-balloon (c). Notice the relatively smooth curve of the balloon catheter

**Figure 11:**
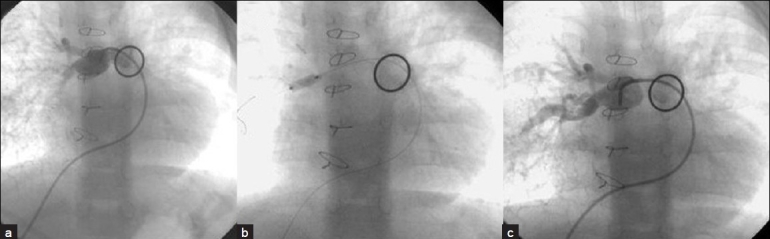
Cutting balloon is being used in the right pulmonary artery. Pre- (a) during (b) and post-balloon (c) angiograms are demonstrated

Using the transhepatic approach, the sheath and catheter are directed posteriorly and leftward toward the atrial septum. Thus, entering the right ventricle for pulmonary artery interventions may require special catheters. Generally, a Judkins right-type catheter with floppy wire or custom-made 45 degree sheaths (RCFW-6.0-35-RB-MUOM-1-294) (Cook, Blomington, IN) would facilitate transhepatic catheter manipulation into the right ventricle and the pulmonary artery [Figure 9]. A balloon-directed catheter may also be used. Once the catheter is in the right ventricle, advancing the wire to the pulmonary arteries is not very different from routine cardiac catheterization. We prefer the Judkins right-type catheters to direct the wire to the intended branches. The balloon catheter course is usually smoother in the left pulmonary artery [Figure 10] when compared to the right pulmonary artery [Figure 11].

### Closure of the tract

**Figure 12:**
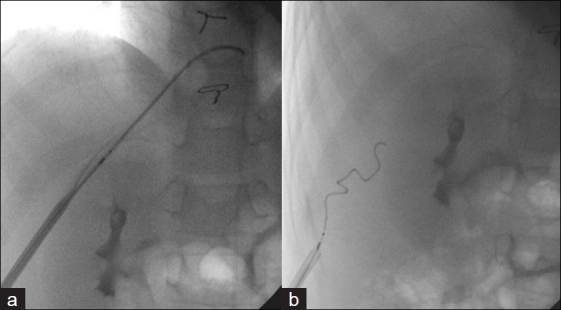
A hand injection through the side arm of the sheath (a) outlines the entry point of the tract. This allows accurate coil placement (b) to occlude the tract

Maintaining hemostasis in the rich vascular hepatic parenchyma is important. Some interventionalists have advocated closure of the tract by coils[2,3] or Gel foam (Upjon, Kalamazoo MI).[7] Others have not felt this was necessary.[14] In this series none of the patients underwent closure of the tract. If the tract is to be closed we usually prefer Flipper^®^ detachable coils (Cook, Blomington, IN.) A delivery catheter one French size smaller than the sheath is placed. The sheath is then slowly withdrawn while obtaining a brief angiogram using the side arm of the sheath. This allows accurate identification of the tract and the intended coil position. Subsequently, the catheter is pulled close to the distal end of the tract [Figure 12]. A small part of the coil is deployed to the distal part of the tract and the rest is in the hepatic parenchyma, ensuring that the coil will not embolize once released, especially as the access would have been lost at that point. Alternatively the sheath is pulled slowly till no more blood is aspirated, indicating that the sheath is within the parenchyma and not in the vein, and the sheath is left in that position for 5 minutes. Subsequently, the sheath is removed and pressure is applied to the tract with the hand positioned in the subcostal area, lifting the liver up, compressing the tract against the hepatic parenchyma, and keeping it in that position for 10 minutes. A pressure dressing is then applied. This has been our preferred approach for the majority of our patients. Using this approach we have not had any problems related to sheath removal in these patients. The decision is guided by the size of the sheath, the use of anti-coagulation, the time since heparin administration, if given, the central venous pressure, and the operator preference.

Post-intervention care is similar to regular cardiac catheterization. The patients are watched for evidence of hemodynamic compromise in the regular recovery area and usually discharged home the following day except if otherwise indicated. We do not routinely obtain ultrasound, blood count or liver profile, as this does not generally impact the clinical management,[2,3] except if clinically indicated. Most patients will have subclinical, subcapsular or intraperitoneal bleeding, which is self-limiting.

## RESULTS

We performed a retrospective review of our transhepatic cardiac catheterizations and interventions to assess the branch pulmonary artery interventions. Out of 45 transhepatic catheterizations, 13 interventions were performed to rehabilitate the branch pulmonary arteries (main pulmonary artery and pulmonary valve were excluded, as well as other non-pulmonary procedures) in nine patients. The interventions included, right pulmonary artery balloon angioplasty with regular balloon in 6 patients, cutting balloon in 1 and left pulmonary artery balloon angioplasty in 6. The mean weight was 9.9 Kg ± 3.4. Largest sheath used was 7 Fr. Patients' age ranged from 8 to 86 months (median 23 months). The mean fluoroscopy time was 19.9 minutes +22.6. The fluoroscopy time was not available in 2 patients. The procedure time was 162 ± 43.7 minutes. The procedure time was not available in 1 patient. Placing the wire was guided, as discussed earlier, by using either a Judkin right-type catheter or balloon-directed catheter. Once the wire was in position, advancing the balloons (or the stiff cutting balloon) was not different from the routine femoral vein approach. None of the patients had complications related to the transhepatic catheterization or intervention. The branch pulmonary artery diameter increased from 4.5 ± 2.2 to 7 ± 3 mm

## DISCUSSION

In the increasingly complicated interventional procedures, alternative approaches are sought to overcome the anatomical difficulties encountered in cases of occluded femoral veins as well as in cases of interrupted inferior venous cava with azygous continuation. Although the jugular approach can be considered, it is generally easier to manipulate the catheter and the balloon from the transhepatic approach. As more experience is gained in this approach it may even become the preferred route for some smaller patients requiring larger sheaths to perform pulmonary artery interventions.

Potential complications include significant intra- /retroperitoneal bleeding,[17] sepsis, portal vein thrombosis, pneumothorax, pleural effusion, injury to the gall bladder or hepatic abscess. Fortunately most of these complications are rare, although fatal bleeding has been reported.[18] Attention to the details of the procedure should minimize these complications. The more commonly reported complication is retro- / intraperitoneal bleeding. This can usually be managed medically by administering blood and blood products. The conservative approach has been advocated even for traumatic liver injuries and is preferred over the surgical approach.[19-22]

## CONCLUSION

In the ever evolving world of catheter interventions the transhepatic approach is a valuable addition to facilitate interventional procedures in selected patients. It can be used in cases with occluded femoral veins and may make the procedures easier than the internal jugular or subclavian approach. Even in the presence of patent femoral veins it may allow placing larger sheaths in small children. The procedure is safe and offers an important alternative to the traditional femoral venous approach.
